# Variations in Circulating Active MMP-9 Levels during Renal Replacement Therapy

**DOI:** 10.3390/biom10040505

**Published:** 2020-03-26

**Authors:** Elena Rodríguez-Sánchez, José Alberto Navarro-García, Jennifer Aceves-Ripoll, Judith Abarca-Zabalía, Andrea Susmozas-Sánchez, Teresa Bada-Bosch, Eduardo Hernández, Evangelina Mérida-Herrero, Amado Andrés, Manuel Praga, Mario Fernández-Ruiz, José María Aguado, Julián Segura, Luis Miguel Ruilope, Gema Ruiz-Hurtado

**Affiliations:** 1 Cardiorenal Translational Laboratory, Institute of Research i+12 (imas12), Hospital Universitario 12 de Octubre, 28041 Madrid, Spain; elena.rodsanchez@gmail.com (E.R.-S.); jalbertong@gmail.com (J.A.N.-G.); jen.ace.rip@hotmail.com (J.A.-R.); judithrit@hotmail.com (J.A.-Z.); andreasusmozas@gmail.com (A.S.-S.); hta@juliansegura.com (J.S.); ruilope@icloud.com (L.M.R.); 2 Service of Nephrology, Hospital Universitario 12 de Octubre, 28041 Madrid, Spain; teresa_bada@hotmail.com (T.B.-B.); ehm3871@yahoo.es (E.H.); evameridaherrero@hotmail.com (E.M.-H.); amado.andres@salud.madrid.org (A.A.); mpragat@senefro.org (M.P.); 3 Unit of Infectious Diseases, Hospital Universitario 12 de Octubre, 28041 Madrid, Spain; mario_fdezruiz@yahoo.es (M.F.-R.); jaguadog1@gmail.com (J.M.A.); 4 School of Doctoral Studies and Research, European University of Madrid, 28670 Madrid, Spain; 5 CIBER-CV, Hospital Universitario 12 de Octubre, 28041 Madrid, Spain

**Keywords:** matrix metalloproteinase-9, dialysis, on-line hemodiafiltration, high-flux dialysis, renal replacement therapy, kidney transplantation

## Abstract

Renal replacement therapy (RRT) is complicated by a chronic state of inflammation and a high mortality risk. However, different RRT modalities can have a selective impact on markers of inflammation and oxidative stress. We evaluated the levels of active matrix metalloproteinase (MMP)-9 in patients undergoing two types of dialysis (high-flux dialysis (HFD) and on-line hemodiafiltration (OL-HDF)) and in kidney transplantation (KT) recipients. Active MMP-9 was measured by zymography and ELISA before (pre-) and after (post-) one dialysis session, and at baseline and follow-up (7 and 14 days, and 1, 3, 6, and 12 months) after KT. Active MMP-9 decreased post-dialysis only in HFD patients, while the levels in OL-HDF patients were already lower before dialysis. Active MMP-9 increased at 7 and 14 days post-KT and was restored to baseline levels three months post-KT, coinciding with an improvement in renal function and plasma creatinine. Active MMP-9 correlated with pulse pressure as an indicator of arterial stiffness both in dialysis patients and KT recipients. In conclusion, active MMP-9 is better controlled in OL-HDF than in HFD and is restored to baseline levels along with stabilization of renal parameters after KT. Active MMP-9 might act as a biomarker of arterial stiffness in RRT.

## 1. Introduction

Chronic kidney disease (CKD) is defined as abnormalities of kidney structure and/or function for a period of at least three months, with implications for health [1]. CKD is an irreversible disorder and often progresses to end-stage renal disease (ESRD), which is life-threatening and requires some form of renal replacement therapy (RRT) such as dialysis or kidney transplantation (KT). Since KT is limited by organ availability, dialysis is the most common form of RRT to remove uremic toxins in ESRD. There are two types of dialysis treatments that can be applied to the ESRD patient: peritoneal dialysis and hemodialysis. Hemodialysis can be further classified depending on the membrane modality (convective vs. diffusive). For example, high-flux dialysis (HFD) is based on the use of high diffusion and low convection, whereas on-line hemodiafiltration (OL-HDF) is based on lower diffusion and higher convection, enabling the removal of middle- and larger-molecular weight substances. However, whatever the dialysis strategy used, it is only a temporary solution until the patient can undergo KT.

Patients with CKD, especially ESRD, are regarded as being at high risk of fatal and non-fatal cardiovascular events because cardiovascular and renal disease often have similar origins and risk factors [2]. Cardiovascular risk is particularly high for patients undergoing dialysis [3] because of CKD-associated complications such as atherosclerosis and systemic inflammation [4]. Indeed, this enhanced risk of atherosclerotic cardiovascular events begins from the earliest stages of CKD. Moreover, dialysis-specific risk factors such as mineral bone disorders and excess fluid load induce vascular calcification and hypertension, which in turn induce arterial stiffness [5], a process characterized by a thickening of the arterial wall and a loss of elastin fiber integrity leading to a decrease in elasticity [6]. Arterial stiffness is a predictor of cardiovascular disease in dialysis and KT recipients [7,8], and is associated with graft dysfunction and rejection [9]. Interestingly, vascular thickening tends to diminish after KT [10] and, in fact, KT improves the longevity and quality of life of patients as compared with long-term dialysis treatment [11], although cardiovascular risk remains higher than in the general population [12].

The anomalous turnover of extracellular matrix components favors deleterious vascular remodeling, which is a major mechanism underlying arterial stiffness. Matrix metalloproteinases (MMPs) play a central role in the regulation of both physiological and pathological connective tissue turnover. In particular, the inducible MMP-9, degrades laminin, elastin, and type IV collagen [13], which are the main components of the glomerular basement membrane of the kidney. In fact, the exaggerated activation of MMP-9 has been associated with an abnormal glomerular basement membrane structure and consequent albuminuria escape in treated hypertension [14]. MMP-9 activation is triggered under stress conditions including inflammation and oxidative stress [13] and is consequently upregulated in inflammation-dependent conditions such as hypertension and associated with cardiovascular risk in patients undergoing hemodialysis [15].

Although the use of MMP-9 as a marker of cardiovascular risk has been widely described, there is a paucity of studies examining how RRT affects its activity [16]. MMP-9 can be assessed as total MMP-9 or active MMP-9 levels measured by the MMP-9/tissue inhibitor of MMP (TIMP)-1 ratio, zymography, or solid-phase immunoassays. Total MMP-9 abundance is, however, not a direct measure of its activity, as both active and inactive MMP-9 are considered. Likewise, the indirect measure of active MMP-9 using the MMP-9/TIMP-1 ratio might be inadequate because it considers that TIMP-1, the endogenous inhibitor of MMP-9, interacts completely with MMP-9 in a 1:1 stoichiometry [17]. It is known that reactive oxygen and nitrogen species can interact with both TIMP-1 and MMP-9, blocking endogenous MMP-9 inhibition and activating MMP-9 constitutively [13] and, therefore, assessment of the MMP-9/TIMP-1 ratio as an indicator of MMP-9 activation is not recommended [18], especially in conditions of oxidative stress such as CKD or ESRD.

Given the heterogeneity in MMP-9 measurements and the gap in our knowledge on MMP-9 activity in RRT, the aim of this study was to assess the effect of different types of RRT (HFD, OL-HDF, and KT) on active MMP-9 measured in a direct manner by zymography and enzyme-linked immunosorbent assay (ELISA), as well as by the direct protein interaction between MMP-9 and TIMP-1 using AlphaLISA^®^ technology (PerkinElmer, Waltham, MA, USA).

## 2. Materials and Methods

### 2.1. Study Population

The study included two independent cohorts of patients receiving RRT: 32 dialysis-dependent patients recruited for a cross-sectional analysis between November 2016 and March 2017 [19], and 46 KT recipients recruited for a longitudinal study between November 2014 and June 2016 [20,21]. The exclusion criterion for KT recipients was development of acute rejection in the first year post-KT. Both cohorts of patients were recruited at the Nephrology Unit of the Hospital Universitario 12 de Octubre (Madrid, Spain). Dialysis-dependent patients underwent clinical examination before dialysis, and KT patients underwent clinical examination before transplant. Pulse pressure (PP) was determined as the difference between systolic blood pressure (SBP) and diastolic (DBP) blood pressure [9]. Blood samples were drawn before (pre-) and after (post-) one dialysis session, or in the case of KT at baseline and 7 days, 14 days, 1 month, 3 months, 6 months, and 12 months post-KT. Blood samples were collected in heparin tubes and immediately centrifuged at 2000 rpm for 10 min. Plasma samples were stored at −80 °C until use.

All patients signed informed consent. The study was approved by the local ethics committee in compliance with the guidelines of the Declaration of Helsinki.

### 2.2. Assessment of Active MMP-9

Active MMP-9 was assessed by zymography and ELISA. Zymography was performed under non-reducing conditions using 10% SDS/PAGE containing 0.1% gelatin. Following electrophoresis, gels were incubated in 500 mM Tris, 6 mM CaCl2, and stained with Coomassie Brilliant Blue R-250 (Bio-Rad, Hercules, CA, USA). Digitalized gel images were analyzed with ImageJ software (NIH, Bethesda, MD, USA). Quantification of active MMP-9 was performed with a commercially available ELISA kit (QuickZyme BioSciences, Leiden, The Netherlands). Given the consistency between zymography and ELISA, follow-up of KT patients was exclusively assessed by ELISA at 7 days, 3 months, and 12 months post-KT. In the case of dialysis-dependent patients, active MMP-9 post-dialysis was corrected according to the weight loss during dialysis using the following equation:

(1) $$Cc=\frac{Cpost}{1+\frac{BWpre-BWpost}{0.2*BWpost}}\;,$$

where *Cc* is the corrected concentration post-dialysis, *Cpost* is the concentration post-dialysis, *BWpre* is the body weight pre-dialysis, and *BWpost* is the body weight post-dialysis [22].

### 2.3. Assessment of Total MMP-9, Total TIMP-1, and MMP-9/TIMP-1 Interaction

Quantification of total MMP-9 and TIMP-1 was performed in KT patients at baseline, 7 days, 3 months, and 12 months post-KT using commercial ELISA Quantikine kits (R&D Systems, Minneapolis, MN, USA). MMP-9/TIMP-1 was calculated by dividing the levels of total MMP-9 by the levels of TIMP-1.

AlphaLISA^®^ technology was used to detect the interaction between MMP-9 and TIMP-1 following a published protocol [18]. Briefly, AlphaLISA^®^ acceptor beads (PerkinElmer, Waltham, MA, USA) were conjugated with an anti-MMP-9 antibody (ThermoFisher Scientific, Waltham, MA, USA) and then incubated with plasma samples from KT recipients and a biotinylated anti-TIMP-1 antibody (ThermoFisher Scientific). Streptavidin-coated donor beads were then added to bind the biotinylated anti-TIMP-1 antibody and detect the MMP-9/TIMP-1 interactions. Plates were read on an EnSpire Multimode Microplate Reader (PerkinElmer) using an excitation wavelength of 680 nm and an emission wavelength of 615 nm.

### 2.4. Statistical Analysis

Normality of data was determined with the Kolmogorov–Smirnov test. HFD and OL-HDF groups were compared using unpaired Student’s t-test or the Mann–Whitney U test. Categorical variables were compared with Fisher’s exact test. Pre- and post-dialysis groups were compared using the Wilcoxon signed-rank test, and KT follow-up groups were compared using the Friedman test. Spearman’s rank-order correlation was used to analyze correlations. Results are expressed as mean ± SEM unless otherwise stated, and *p*-values < 0.05 were considered significant. Analyses were performed using GraphPad Prism 6 (GraphPad Software Inc., San Diego, CA, USA) and SPSS Statistics v22 (IBM, Armonk, NY, USA).

## 3. Results

### 3.1. Clinical Characteristics

Baseline characteristics of all hemodialysis patients stratified according to the type of dialysis applied (HDF or OL-HDF) are shown in Table 1. No differences were found in the mean age between the two groups. Likewise, there were no differences between the HFD and OL-HDF groups for hypertension, SBP, PP, diabetes mellitus, or N-terminal pro-brain natriuretic peptides. Dialysis-related parameters were also the same between the groups except for the duration of dialysis. Patients in the OL-HDF group were significantly longer on dialysis (31.4 vs. 98.4 months for HFD and OL-HDF, respectively). There were no differences between the groups for the cause of CKD or the medication used. The proportion of men was significantly higher in the OL-HFD group (65%) than in the HFD group (11%). Baseline characteristics of the KT recipients are shown in Table 2.

### 3.2. Dialysis Reduces Active MMP-9 Levels

**Figure 1:**
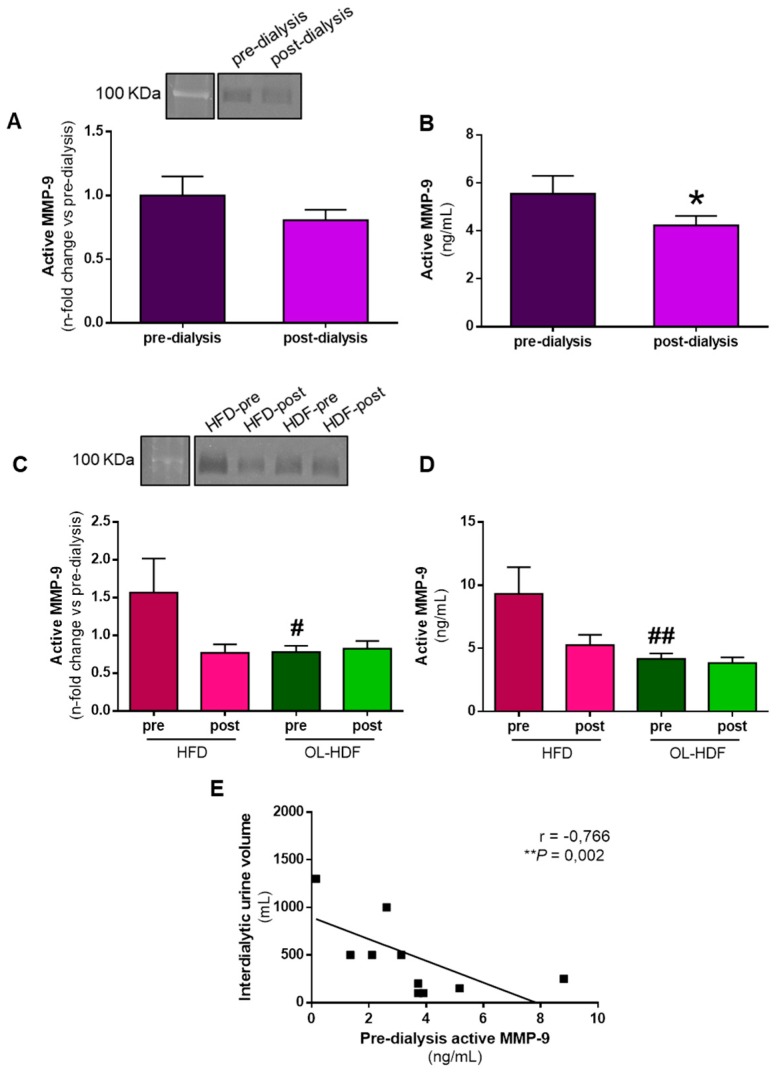
Matrix metalloproteinase (MMP)-9 activity in pre- and post-dialysis according to the type of dialysis applied. (**A**) Representative zymography gel showing plasma gelatinase MMP-9 activity (upper panel) and quantification (bottom panel). (**B**) Quantification of active MMP-9 by ELISA in plasma of dialysis patients before (pre-dialysis) and after (post-dialysis) one session of dialysis. (**C**) Representative zymography gel showing plasma gelatinase MMP-9 activity (upper panel) and quantification (bottom panel). (**D**) Quantification of active MMP-9 formed by ELISA in plasma of dialysis patients stratified as those on high-flux dialysis (HFD) (pre- and post-dialysis) and on-line hemodiafiltration (OL-HDF) (pre- and post-dialysis). (**E**) Negative correlation between interdialytic urine volume and pre-dialysis active MMP-9 in dialysis patients with residual urine volume. Correlation was performed using Spearman’s test and a linear regression of the data is displayed. * *p* < 0.05 vs. pre-dialysis; ^#^ *p* < 0.05 and ^##^ *p* < 0.01 vs. HFD pre-dialysis.

We first compared the plasma levels of active MMP-9 in all dialysis patients pre- and post-dialysis. Active MMP-9 estimated by zymography (Figure 1A) or quantified by ELISA (Figure 1B) was lower post-dialysis than pre-dialysis, and this was significant for the ELISA analysis. Baseline plasma levels of active MMP-9 were significantly lower in the OL-HDF group than in the HFD group, both by zymography (Figure 1C) and ELISA (Figure 1D), and active MMP-9 levels were reduced by dialysis only in the HFD group while OL-HDF patients’ active MMP-9 levels remained at the same level after the dialysis process. Active MMP-9 negatively correlated with the interdialytic urine volume (Figure 1E), in the sense that less urine volume was associated with higher MMP-9 activity.

### 3.3. Kidney Transplantation Increases Total and Active MMP-9 and Total TIMP-1 Levels, but Not MMP-9:TIMP-1 Protein Interactions

**Figure 2:**
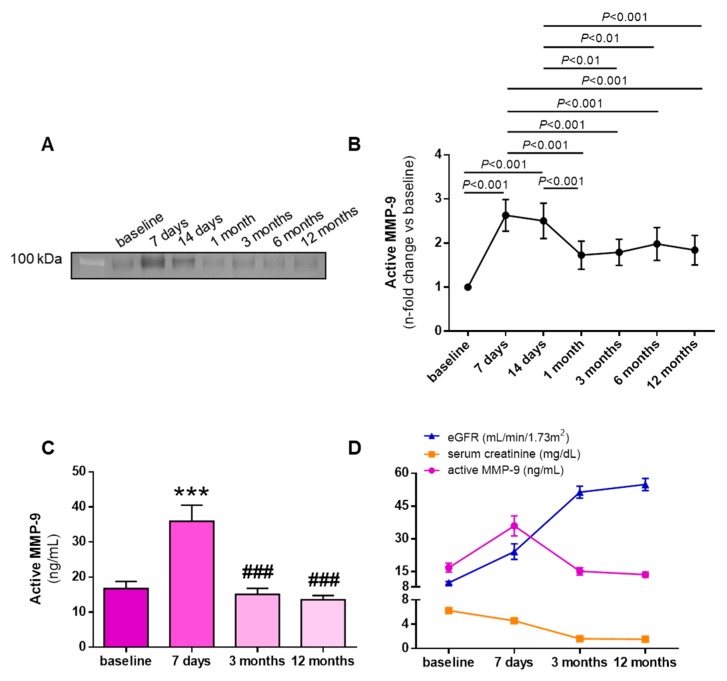
MMP-9 activity profile before and after kidney transplantation. (**A**) Representative zymography gel showing plasma gelatinase MMP-9 activity. (**B**) Quantification of active MMP-9 by zymography in plasma of kidney transplantation (KT) patients at baseline (before KT) and after 7 and 14 days, and 1, 3, 6, and 12 months. (**C**) Quantification of active MMP-9 by ELISA in plasma of KT patients at baseline and after 7 days, 3 months, and 12 months. (**D**) Evolution of eGFR, plasma creatinine, and active MMP-9 at baseline and 7 days, 3 months, and 12 months after KT. *** *p* < 0.001 vs. baseline and ^###^ *p* < 0.001 vs. 7 days after KT.

Representative gel zymography of active MMP-9 in KT recipients before KT and at follow-up is shown in Figure 2A. Active MMP-9, measured by gel zymography (Figure 2B) or quantified by ELISA (Figure 2C), increased significantly 7 days after KT. Active MMP-9 levels remained high 14 days after KT (Figure 2A,B), but thereafter decreased significantly at one month after KT, reaching levels not significantly different to baseline at 3 and 12 months after KT (Figure 2A–C). Active MMP-9 levels were related to renal function, decreasing as estimated glomerular filtration rate (eGFR) increased and plasma creatinine decreased (Figure 2D).

**Figure 3:**
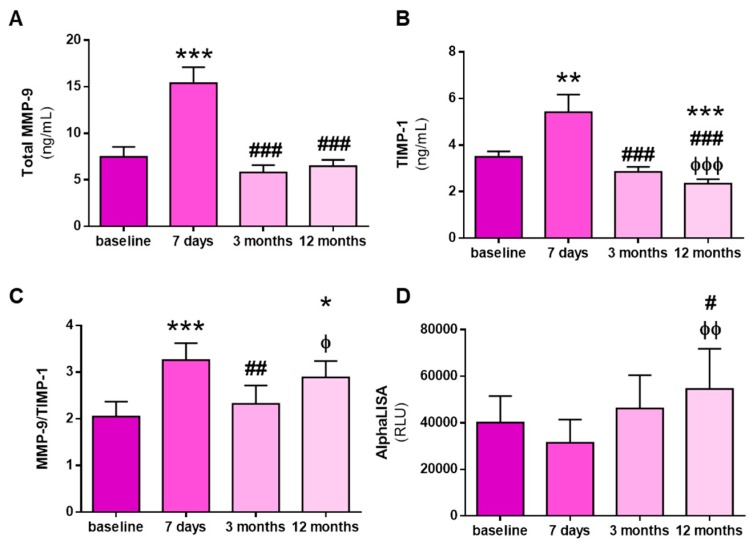
Total protein MMP-9 and tissue inhibitor of MMP (TIMP)-1 levels, MMP-9/TIMP-1 ratio, and MMP-9:TIMP-1 interactions before and after KT. (**A**) Total MMP-9 protein levels (**B**) and total TIMP-1 protein levels quantified by ELISA at baseline and at 7 days, 3 months, and 12 months after KT. (**C**) MMP-9/TIMP-1 ratio estimation. (**D**) AlphaLISA^®^ MMP-9:TIMP-1 interaction immunoassay expressed as binding relative luminescence units (RLUs). * *p* < 0.05, ** *p* < 0.01, *** *p* < 0.001 vs. baseline; ^#^ *p* < 0.05, ^##^ *p* < 0.01 and ^###^ *p* < 0.001 vs. 7 days after KT; and ^φ^ *p* < 0.05, ^φφ^ *p* < 0.01, ^φφφ^ *p* < 0.001 vs. 3 months after KT.

To evaluate whether the changes in active MMP-9 abundance were due to changes in its expression or, alternatively, to its endogenous inhibition by TIMP-1, we measured total MMP-9 and TIMP-1 levels in KT recipients. Total MMP-9 levels increased significantly at 7 days after KT relative to baseline levels, but significantly decreased at 3 and 12 months after KT (Figure 3A). A similar pattern was observed for TIMP-1 levels, with a significant increase at 7 days after KT and a return to baseline levels at 3 months (Figure 3B). However, TIMP-1 levels continued to decrease at 12 months after KT and were significantly lower than baseline levels at this time (Figure 3B). To indirectly estimate MMP-9 activity and following the line of the majority of clinical studies, we calculated the MMP-9/TIMP-1 ratio, which revealed a significant increase in the MMP-9/TIMP-1 ratio over baseline levels at 7 days after KT (Figure 3C). The ratio decreased to baseline levels 3 months after KT but increased 12 months after KT, reaching an intermediate level (Figure 3C). To confirm the interaction between both molecules, we used the AlphaLISA protocol to measure MMP-9:TIMP-1 protein interactions [18]. No changes in MMP-9:TIMP-1 interactions were found until 12 months after KT, when the interaction was significantly higher than at baseline (Figure 3D).

### 3.4. Active MMP-9 Positively Correlates with Arterial Stiffness in RRT Patients, but Not with Systolic Blood Pressure

**Figure 4:**
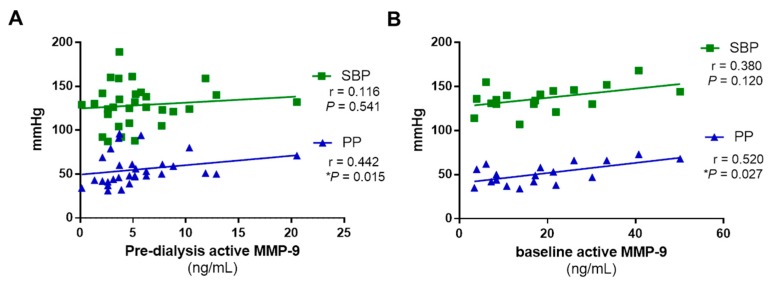
Correlation between MMP-9 activity and systolic blood pressure (SBP) and pulse pressure (PP) at pre-dialysis and before KT. (**A**) No association of MMP-9 activity with SBP (in green) and a positive significant correlation with PP (in blue) in dialysis patients in pre-dialysis. (**B**) No association of MMP-9 activity with SBP (in green) and a positive significant correlation with PP (in blue) in KT recipients before KT (baseline). Correlation was performed using Spearman’s test and a linear regression of the data is displayed.

We examined for correlations between active MMP-9 levels, SBP, and the arterial stiffness PP parameters in patients undergoing RRT. No correlation was found between active MMP-9 and SBP in the dialysis or KT cohorts (Figure 4A,B). However, we found a positive significant correlation between active MMP-9 levels and PP in both dialysis and KT cohorts (Figure 4A,B).

## 4. Discussion

Our study shows that: (i) patients undergoing OL-HDF have lower levels of active MMP-9 compared with those undergoing HFD; (ii) active MMP-9 increases early post-KT at 7 and 14 days and stabilizes in parallel with renal markers at 3 months after KT; and (iii) active MMP-9 is associated with arterial stiffness measured by PP in RRT (pre-dialysis and before KT).

Renal dysfunction is characterized by a uremic state that aggravates as renal function declines, reaching its maximum in ESRD. This state is intimately associated with all-cause mortality and especially with cardiovascular morbidity and mortality. Undoubtedly, RRT improves the lifespan and the quality of life of patients with ESRD. Among RRT patients, KT significantly improves the quality of life compared with dialysis, but the access to renal transplants is limited and not all ESRD patients can undergo a surgical procedure. Although dialysis is the cornerstone of RRT, it is also associated with a state of chronic inflammation and oxidative stress [23]. Dialysis *per se* triggers an increase in oxidative stress and inflammation because of incompatibilities with the dialysis membrane, which activates circulating leukocytes, and also because low-molecular weight antioxidant systems are filtered and therefore eliminated during the procedure. In addition, the type of dialysis used directly influences the oxidative status of the patients. Indeed, recent studies demonstrate that patients under OL-HDF have less inflammation, endothelial damage, and oxidative stress [19,24,25], which could be associated with the improved prognosis in this group [26,27]. The reduction in middle-molecules is associated with the preservation of residual renal function [28], which decreases the mortality risk in dialysis [29] and is improved in OL-HDF [30]. The systemic activation of MMP-9 is a marker of a lasting inflammatory state that is inherent to renal disease, as has been demonstrated even with adequate pharmacological treatment in patients with CKD [14,31]. In the case of ESRD, and especially dialysis, there are conflicting results on the levels of MMP-9 in the pre- and post-dialysis states [32,33]. This could be due to the different types of dialysis membranes [34,35], or to the type of dialysis itself [19,25]. We demonstrate herein that active MMP-9 is cleared during dialysis when the type of dialysis is not considered, but a decrease in active MMP-9 is only seen in HFD. Other authors have described a decrease in total MMP-9 during OL-HDF, but not during other hemodialysis [36]. Nevertheless, total MMP-9 does not necessarily correlate with active MMP-9, and the measurement of active MMP-9 is a more direct assessment of its pathophysiological activity [14]. The fact that only HFD effectively clears active MMP-9 might suggest that HFD is more efficient than OL-HDF; however, pre-dialysis active MMP-9 was significantly lower in the OL-HDF group than in HFD patients, indicating that the levels of active MMP-9 are better controlled in patients under OL-HDF. Moreover, residual renal function is associated with lower inflammation in OL-HDF [37,38]. Supporting this, we also observed that active MMP-9 is lower in patients with greater interdialytic urine volume, which is superior to eGFR as an estimate of residual renal function in dialysis [28].

KT is the only curative treatment for ESRD and long-term survival is significantly greater after KT than after dialysis. Despite its benefits, however, KT is not without risks, such as the inevitable ischemia and reperfusion (I/R) injury, which triggers several processes such as immune system activation, endothelial dysfunction, and cell death. Likewise, I/R induces morphological changes in the kidney that lead to fibrosis, as shown by the elevation of fibrosis-related biomarkers in the kidney of patients with chronic allograft nephropathy [39]. By contrast, a well-known benefit of KT is the normalization of inflammatory and oxidative stress markers [40]. For example, the C-terminal agrin fragment, a biomarker for kidney function and a breakdown product of agrin, the major proteoglycan of the glomerular basement membrane, strongly correlates with creatinine and eGFR and is stabilized at 1–3 months after KT [41]. In the same line, interleukin 6, a well-known precursor of MMP-9 activation, shows a burst of activity one week after KT before stabilizing [42]. In the present study, we demonstrate that systemic active MMP-9 levels increase one week after KT, but rapidly and significantly decrease from the second week after surgery. We speculate that the increase in active MMP-9 evident one week after KT likely originates from neutrophils in response to leukocyte activation inherent to I/R processes. This is supported by experimental studies demonstrating that MMP-9 promotes mononuclear cell infiltration in a rat model of early allograft nephropathy [43].

The peak in active MMP-9 observed early post-KT (after 7 and 14 days) might be due to an increase in total MMP-9 or an enhancement in the enzyme activation by the inflammatory environment. MMP-9 is synthesized as a zymogen with a covered zinc-containing active site. Other MMPs and MMP-9 itself cleave the peptide chain that covers the active site in physiological conditions. However, reactive oxygen and nitrogen species can pathologically expose the active site by binding to the zinc ion. Reactive oxygen and nitrogen species can also bind to TIMP-1 and hamper the physiological inhibition of MMP-9. Indeed, MMP-9:TIMP-1 interactions are reduced under conditions of increased oxidative stress and can therefore mask the results obtained from the surrogate marker of MMP-9 activity, the MMP-9/TIMP-1 ratio [18]. Given the increase in oxidative stress associated with I/R, we aimed to investigate whether the peak in active MMP-9 was due to the pathological activation of MMP-9 or to an increase in MMP-9 protein levels. Our results indicate that the peak in active MMP-9 was in fact due to an increase in total MMP-9 expression, and the results of the MMP-9/TIMP-1 ratio indicate the same. However, the reduction in TIMP-1 levels at 12 months post-KT leads to an increase in the MMP-9/TIMP-1 ratio, erroneously suggesting increased MMP-9 activity. Indeed, active MMP-9 is unaltered 12 months post-KT because the MMP-9:TIMP-1 interaction is increased, supporting the importance of measuring active MMP-9 rather than the MMP-9/TIMP-1 ratio. The suggested increase in active MMP-9 by the MMP-9/TIMP-1 ratio indicates an increase in mortality risk [44]. However, total and real active MMP-9 do not vary, and TIMP-1, which is also associated with mortality [45], is reduced. Therefore, the patients might actually have a reduced risk of mortality as the KT becomes more stable.

Finally, arterial stiffness increases SBP by wave reflection and decreases DBP, which increases PP, a marker of large artery stiffness [46]. Arterial stiffness is attenuated after KT as compared with hemodialysis [9,47,48,49,50], and is subject to donor age, living kidney donation, and mean blood pressure [5]. Our study shows for the first time, to our knowledge, that active MMP-9 correlates with PP in patients treated with RRT. We found that, independently of the type of dialysis, PP is lower as the levels of active MMP-9 decline. A decrease in active MMP-9 suggests that arterial stiffness might be reduced in patients undergoing OL-HDF. However, we failed to find differences in PP between groups of patients on the different types of dialysis, in accord with results from other groups describing that arterial stiffness is not different between hemodialysis and HDF [5]. We also found that baseline active MMP-9 correlates with PP before KT, supporting the idea that a reduction in active MMP-9 levels in ESRD patients is needed to ameliorate arterial stiffness.

The main limitation of the present study was the small population of dialysis patients. More studies are needed in order to confirm our results, but this pilot study is the first step in assessing the insights of arterial stiffness in different types of dialysis.

## 5. Conclusions

Active MMP-9 is lower in OL-HDF than in HFD and shows a peak in KT recipients after I/R in the early post-KT stages (7 and 14 days). However, the peak in active MMP-9 is resolved in parallel with the restoration of renal function, indicating that MMP-9 activity is likely a surrogate of I/R injury. In both contexts—dialysis and KT—active MMP-9 is associated with PP in pre-dialysis and just before KT, indicating that MMP-9 is a marker of vascular dysfunction in patients undergoing RRT.

## Figures and Tables

**Table 1:** Baseline characteristics of dialysis patients.

	All Patients (*n* = 32)	HFD Patients (*n* = 9)	OL-HDF Patients (*n* = 23)	*p*-Value
Age (years)	60.0 ± 16,4	64.7 ± 22.0	58.2 ± 13.8	0.322
Male sex (*n*, %)	16 (50)	1 (11)	15 (65)	0.016
Hypertension (*n*, %)	25 (78)	7 (78)	18 (78)	0.999
SBP (mmHg)	128.4 ± 23.0	126.2 ± 23.2	129.3 ± 23.4	0.739
PP (mmHg)	55.5 ± 17.5	58.4 ± 14.6	54.3 ± 18.7	0.556
Diabetes mellitus (*n*, %)	7 (22)	2 (22)	5 (22)	0.999
NT-proBNP (pg/mL)	2565 ± 1735	2753 ± 1245	2509 ± 1881	0.769
Dialysis-related parameters				
Serum creatinine (mg/dL)	7.61 ± 2.22	7.16 ± 2.22	7.78 ± 2.25	0.486
Serum albumin (g/dL)	4.05 ± 0.43	3.91 ± 0.24	4.10 ± 0.48	0.259
Potassium (mEq/L)	5.15 ± 0.90	5.06 ± 0.78	5.19 ± 0.95	0.725
Bicarbonate (mEq/L)	21.3 ± 2.9	21.2 ± 3.8	21.3 ± 2.6	0.974
Dialysis vintage (months)	79.5 ± 74.3	31.4 ± 24.5	98.4 ± 79.1	0.020
Kt/V	1.63 ± 0.24	1.68 ± 0.25	1.61 ± 0.24	0.463
eGFR (ml/min/1.73 m^2^)	6.92 ± 3.00	6.48 ± 2.92	7.09 ± 3.08	0.614
Residual diuresis (*n*, %)	15 (52)	5 (56)	10 (43)	0.699
Interdialytic urine volume (mL)	375 (163–875)	250 (100–500)	500 (175–1000)	0.518
Cause of CKD				0.547
Glomerulonephritis (*n*, %)	7 (22)	2 (22)	5 (22)	
Diabetic nephropathy (*n*, %)	5 (16)	1 (11)	4 (17)	
Polycystic kidney disease (*n*, %)	4 (12)	0 (0)	4 (17)	
Hypertensive nephropathy (*n*, %)	2 (6)	0 (0)	2 (9)	
Other or undetermined (*n*, %)	14 (44)	6 (67)	8 (35)	
Medication				
ACEi/ARB (*n*, %)	8 (25)	2 (22)	6 (26)	0.999
Diuretics (*n*, %)	3 (9)	0 (0)	3 (13)	0.541
β-blockers (*n*, %)	13 (41)	5 (56)	8 (35)	0.427
Cinacalcet (*n*, %)	3 (9)	0 (0)	3 (13)	0.541
Paricalcitol (*n*, %)	12 (38)	5 (56)	7 (30)	0.253

SBP: systolic blood pressure; PP: pulse pressure; NT-proBNP: N-terminal (NT)-pro B-type natriuretic peptide; Kt/V: where K is the dialyzer urea clearance, t is the total treatment time, and V is the total volume within the body that urea is distributed; eGFR: estimated glomerular filtration rate; CKD: chronic kidney disease; ACEi: angiotensin converting enzyme inhibitor; ARB: angiotensin receptor blocker.

**Table 2:** Baseline characteristics of kidney transplant recipients.

	All Patients (*n* = 46)
Age of the recipient (years)	54.6 ± 15.8
Male sex (*n*, %)	27 (59)
Previous kidney transplant (*n*, %)	4 (9)
Pretransplant dialysis (*n*, %)	41 (89)
Dialysis vintage (months)	21.0 ± 19.0
SBP (mmHg)	134.5 ± 14.8
PP (mmHg)	49.9 ± 12.9
Serum creatinine (mg/dL)	6.27 ± 2.56
Serum albumin (g/dL)	4.23 ± 0.52
eGFR (mL/min/1.73 m^2^)	9.82 ± 4.28
Donor	
Male sex (*n*, %)	25 (54)
Age (years)	52.6 ± 14.6
Living donor (*n*, %)	5 (11)
Number of HLA-mismatches	5 (4–5)
Cause of CKD	
IgA nephropathy	9 (20)
Glomerulonephritis	2 (4)
Diabetic nephropathy	7 (15)
Polycystic kidney disease	8 (17)
Hypertensive nephropathy	3 (7)
Other or undetermined	17 (37)
Induction therapy	38 (83)
Maintenance immunosuppression	
Steroids	46 (100)
Tacrolimus	46 (100)
Mycophenolate mofetil/mycophenolic acid	42 (91)
Cyclosporine A	0 (0)

SBP: systolic blood pressure; PP: pulse pressure; eGFR: estimated glomerular filtration rate; CKD: chronic kidney disease; HLA: human leukocyte antigen.
